# Round about the Asylums

**Published:** 1888-02-25

**Authors:** 


### Round About the Asylums.—I.

By a Medical Superintendent.

### AMERICAN REPORTS.

It is probable that few of our readers ever see the annual reports of the various lunatic asylums, and fewer still would have patience to pore over the letterpress and tables of which they are composed. These reports vary but little from year to year. The circumstances under which they are written are the same, the subjects under notice are the same, and hence the details are very similar. Each report may be said to consist of five parts: (1) The report of the Committee to Quarter Sessions, (2) the report of the Medical Superintendent, (3) the report of the Commissioners in Lunacy as to the state of the asylum on the day of their visit, (4) the medical tables, and (5) the financial statements.

All English asylums begin their years with January 1st, and end with December 31st; but many of the American asylums reckon from October 1st to September 30th. It thus happens that our cousins' reports find their way to the editor's room before our own are in print. It is pretty generally admitted that the American asylums are inferior to the English in matters of comfort, and in the amount of freedom enjoyed by the patients; while they are superior to ours in the number of medical officers, and, presumably therefore, in the amount of direct medical supervision of the patients. Whether an increase in the medical staff means an increase of medical work done is a matter with which we are not now concerned. We notice that many of the American asylums have lady physicians for the women's side of the asylums, and this is so manifestly a step in the right direction that we venture to hope it is an example which will soon be followed in all our large county asylums.

### STATE LUNATIC HOSPITAL AT HARRISBURG, PENN.

This asylum, or hospital, has just issued its thirty-seventh annual report. It contains 497 patients, and there are five medical officers, two of whom are ladies. In Pennsylvania the same difficulty seems to exist as in so many English counties—want of accommodation for the class between paupers and those able to pay the charges in private asylums. The hospital is now being enlarged, and when the additions are ready for occupation there will be more room for these people whose necessities Dr. Gerhard evidently fully recognises. The numbers increased during the year by 36, and Dr. Gerhard makes the observation that if we wish to prevent the accumulation of chronic cases we must aim at prevention rather than cure. Very true. But how is it to be done? There's the rub. "We know that insanity is a disease, and as such amenable to treatment. It has been believed by many that insanity was more curable than other diseases of an equally serious type. Under the influence of this idea, magnificent buildings have been erected, which have been called hospitals, not asylums, because they were intended as curative institutions. But, within a few years, we have come to look upon insanity as not so curable, and the tendency is to make cheaper provision and to increase the capacity of hospitals." Here, again, the New Country and the Old are at one. 73 patients were admitted during the year; 31 were discharged recovered, and 44 died.

### LUNATIC HOSPITAL, NORTHAMPTON, MASS.

This is the hospital lately so ably superintended by Dr. Pliny Earle, a gentleman well known in English asylums, not only by his writings on insanity, but also by his visits to our county asylums. He was succeeded by his former assistant, Dr. Nimms, who, in 1885, made the tour of English asylums, in which he was last year followed by his assistant, Dr. Hall. There were on the last day of the fiscal year 469 patients. During the year there were 76 admissions, 27 discharged recovered, and 31 deaths. Regarding criminal lunatics, the trustees, or visitors as we would call them, made the following very sensible remarks: "The plan of placing the criminal and vicious classes of the insane in an institution by themselves is, in the opinion of the trustees of this hospital, a move in the right direction. They are always a disturbing element in the wards of a hospital, both in their habits and in their resistance to good order and discipline. In the present arrangement of this hospital they are necessarily brought in contact with other patients. It is no more just to compel the insane of good character to associate with them than it would be the sane." Over and over again the same complaint has emanated from English asylums, but nothing has been done, and nearly every one of our county and borough asylums has its little band of criminals. Dr. Nimms' report covers a wide area, and deals with many important questions, but want of space forbids extracts. The weekly charge for State patients is three and a-half dollars, or about 14*s.* 8*d.* of our money.

### LUNATIC HOSPITAL, TAUNTON, MASS.

During the year this hospital contained a daily average of 637 patients. There are five medical officers. This must be looked upon as an ample staff. 271 patients were admitted during the year; 59 were discharged recovered, and 59 died. Dr. Brown mentions the use of the hydrobromate of hyoscine in excitement in doses of one-fiftieth and one-hundredth of a grain. Many of his first trials were unsatisfactory, but subsequently better results were obtained, and he says: "Our experience of it would indicate it to be a valuable remedy in certain cases when properly used." Regarding restraint, he writes: "If complete non-restraint were desirable to gratify an abstract idea or sentiment, it could easily be accomplished at some risk to the personal safety and comfort of a few patients." It is always possible to ride a hobby too hard; but it has certainly been found practical in English asylums to abolish restraint, except in surgical cases; and while English superintendents do not hesitate to order restraint where necessary, they still hold that restraint should form no part of the asylum armamentarium. It is doubtful whether any asylum in England possesses a strait jacket. When speaking of occupation, Dr. Brown says: "The male patients, as a rule, are more willing to work, and—which would hardly be expected—the male attendants receive more assistance in the ward work from the men than do the female attendants from the women." Our experience in English asylums fully bears this out.

### THE STATE ASYLUM, BUFFALO.

The highest number resident was 404. There were during the year 318 admissions, 107 recoveries, and 44 deaths. The report of the trustees runs on to ten pages. They detail an interesting case of "broken ribs, which involved an action against the attendants," who were, however, acquitted; and there can be no doubt that the acquittal was right. We are pleased to quote the following from Dr. Andrews' report: "For some months past some of the doors of the patients' rooms in the most quiet wards have been left unlocked at night. This gives them an opportunity to visit the service rooms, if necessary, relieves the mind of fear in regard to danger from fire, and by the trust imposed increases the confidence and contentment. No injurious consequences nor influences have so far been experienced from the change, nor has anyone taken advantage of the increased liberty allowed." It would seem that 75 per cent. of the men and 74 per cent. of the women were usefully employed. This is the highest percentage we have yet met with in American reports, and, indeed, very few English ones exceed it. Not fewer than 26 per cent. of the patients are allowed to go beyond the grounds on parole.

